# *In Silico* Identification of Potential Sites for a Plastic-Degrading Enzyme by a Reverse Screening through the Protein Sequence Space and Molecular Dynamics Simulations

**DOI:** 10.3390/molecules27103353

**Published:** 2022-05-23

**Authors:** Krit Charupanit, Varomyalin Tipmanee, Thana Sutthibutpong, Praopim Limsakul

**Affiliations:** 1 Department of Biomedical Sciences and Biomedical Engineering, Faculty of Medicine, Prince of Songkla University, Songkhla 90110, Thailand; krit.ch@psu.ac.th (K.C.); tvaromya@medicine.psu.ac.th (V.T.); 2 Theoretical and Computational Physics Group, Department of Physics, Faculty of Science, King Mongkut’s University of Technology Thonburi (KMUTT), Bangkok 10140, Thailand; thana.sut@kmutt.ac.th; 3 Center of Excellence in Theoretical and Computational Science (TaCS-CoE), Faculty of Science, King Mongkut’s University of Technology Thonburi (KMUTT), Bangkok 10140, Thailand; 4 Division of Physical Science, Faculty of Science, Prince of Songkla University, Songkhla 90110, Thailand; 5 Center of Excellence for Trace Analysis and Biosensor (TAB-CoE), Faculty of Science, Prince of Songkla University, Songkhla 90110, Thailand

**Keywords:** alanine scanning mutagenesis, molecular docking, PETase, molecular dynamics, site-saturation mutagenesis

## Abstract

The accumulation of polyethylene terephthalate (PET) seriously harms the environment because of its high resistance to degradation. The recent discovery of the bacteria-secreted biodegradation enzyme, PETase, sheds light on PET recycling; however, the degradation efficiency is far from practical use. Here, *in silico* alanine scanning mutagenesis (ASM) and site-saturation mutagenesis (SSM) were employed to construct the protein sequence space from binding energy of the PETase–PET interaction to identify the number and position of mutation sites and their appropriate side-chain properties that could improve the PETase–PET interaction. The binding mechanisms of the potential PETase variant were investigated through atomistic molecular dynamics simulations. The results show that up to two mutation sites of PETase are preferable for use in protein engineering to enhance the PETase activity, and the proper side chain property depends on the mutation sites. The predicted variants agree well with prior experimental studies. Particularly, the PETase variants with S238C or Q119F could be a potential candidate for improving PETase. Our combination of *in silico* ASM and SSM could serve as an alternative protocol for protein engineering because of its simplicity and reliability. In addition, our findings could lead to PETase improvement, offering an important contribution towards a sustainable future.

## 1. Introduction

Polyethylene terephthalate (PET) is the most widely used thermoplastic polymer in daily life. The polymerization of ethylene glycol (EG) and terephthalic acid (TPA) through the ester bond causes PET to be resistant to natural degradation due to its chemical inertness; thus, the overwhelming accumulation of PET leads to serious environmental problems [1]. Although mechanical and chemical processes are common methods to recycle and depolymerize PET, these processes consume high energy and resources [2,3,4]. Biodegradation by microorganism-derived enzymes extracted from bacteria or fungi has been successfully employed in various fields such as oil spill bioremediation and municipal solid wastes [5,6,7,8,9]. Therefore, microorganism-derived enzymes that naturally break down PET could become a promising alternative approach for recycling PET.

PET-degrading enzymes have been discovered in many microorganisms, such as TfCut2 from the thermophilic bacterium *Thermobifida fusca* [10], Cut190 from *Saccharomonospora viridis* AHK190 [11], and LCC identified from the leaf-branch compost metagenome [12]. In addition, these enzymes have been further optimized and modified by protein engineering [13]. For example, introducing a disulfide bond (D204C/E253C) at the calcium binding site [14] or mutations to remodel the binding groove of TfCut2 [15,16], introducing mutations (S226P, S226P/R228S) to increase Ca^2+^-binding sites on the protein surface of Cut190 [11,17], and introducing a disulfide bond (D238C/S283C) of LCC [18], showed an improvement of the enzyme catalytic efficiency and stability. However, the uses of these enzymes are limited because of their low enzymatic activities [19,20]. Recently, the more applicable enzyme system, secreted from *Ideonella sakaiensis* 201-F6, has gained much attention due to its high degradation efficiency at a mild temperature [21]. This system relies on two enzymes, PETase and MHETase, that synergistically depolymerize PET. While PETase initially digests PET, producing mono(2-hydroxyethyl) terephthalate acid (MHET) and secondary products such as TPA and bis(2-hydroxyethyl)-TPA, MHETase later digests and converts MHET into the monomer such as TPA and EG [21,22]. PETase can degrade highly crystalline PET (e.g., PET film) at a rate of 0.13 mg/cm^2^ per day at 30 °C [21]. Although PETase showed better PET-degrading activity than other enzymes at near room temperature, its degradation rate needs to be further improved to be used in the recycling industry.

Understanding the binding mode of PET to the PETase catalytic site is crucial to improve the enzymatic activity. Crystal structures of PETase have been determined [22,23,24,25,26], which allow researchers to rationally modify PETase with better performance. Consisting of seven alpha helices and nine beta strands with Ser–His–Asp as a catalytic triad, PETase belongs to the α/β hydrolase superfamily that can hydrolyze PET [23,27]. However, due to difficulties in co-crystallization and the low solubility of the PET polymer, the PETase structure in complex with PET is still unavailable [23,25]. As a result, only computational approaches, such as molecular docking and molecular dynamics (MD) (i.e., quantum mechanics/molecular mechanics (QM/MM) and a density functional theory (DFT)-based QM/MM) have been employed to investigate their binding mode and molecular interactions [22,23,26,28,29,30]. Although the proposed PETase catalytic mechanisms from these studies are similar in that PET depolymerization occurs at the active site of PETase (S160, H237, and A206) with the help of other hydrophobic residues around the binding cleft that brings the carbonyl carbon atom of PET into close contact for nucleophilic attack from S160, the reaction mechanism of Ser–His–Asp-initiated nucleophilic attack is still inconclusive.

Several PETase mutations have been proposed to improve PETase activity through rational protein engineering, including those with one mutation site (e.g., R280A [23], Y87A [24], or I208F [31]) and two mutation sites (e.g., W159H/S238F [22], S121E/D186H [32]). However, the practical application of these variants in the recycling industry has yet to be reported. Protein engineering-based rational design is commonly used to identify potential enzyme mutation sites. Then, the enzymatic activity of an individual variant in a small variant library generated by site-directed mutagenesis is characterized, which is easier to handle than a large and diverse library of protein variants generated by random mutagenesis in directed evolution [33,34]. However, the small size of the library and the simpler protein space may restrict the possibility of discovering improved protein variants. To address this, semi-rational design, which combines rational design and directed evolution approaches, can generate a small but diverse library using the site-saturation mutagenesis (SSM) technique to increase the activity of PET-degrading enzymes [18,35].

A simple and systematic approach is introduced to identify the potential mutation site of PETase without performing laborious experimental screening of desired variants from the large protein sequence space generated by SSM. In this work, the PETase structure [23] and the PET tetramer, which represents the PET polymer, were used to identify the position and potential PETase mutation sites using combined multiple sequence alignment (MSA) [36,37], *in silico* alanine scanning mutagenesis (ASM) [38], and computational protein–ligand simulation [39]. *In silico* SSM was then used to explore the protein sequence space of PETase for the PETase-PET complex [40]. The PETase sequence space was constructed for the first time to elucidate the structural significance of potential PETase mutations in order to improve PET-degrading activity. Finally, MD simulations were used to investigate the interactions between selected PETase variants and PET. The majority of our predicted PETase variants agreed well with experimental studies, while the remaining variants are novel. As a result, our protocol could be used as an alternative approach to predict the mutation site of enzymes other than PETase. Furthermore, our findings can be used to improve the PET-degrading activity of PETase.

## 2. Results

### 2.1. Search for Candidate Mutation Sites

**Figure 1:**
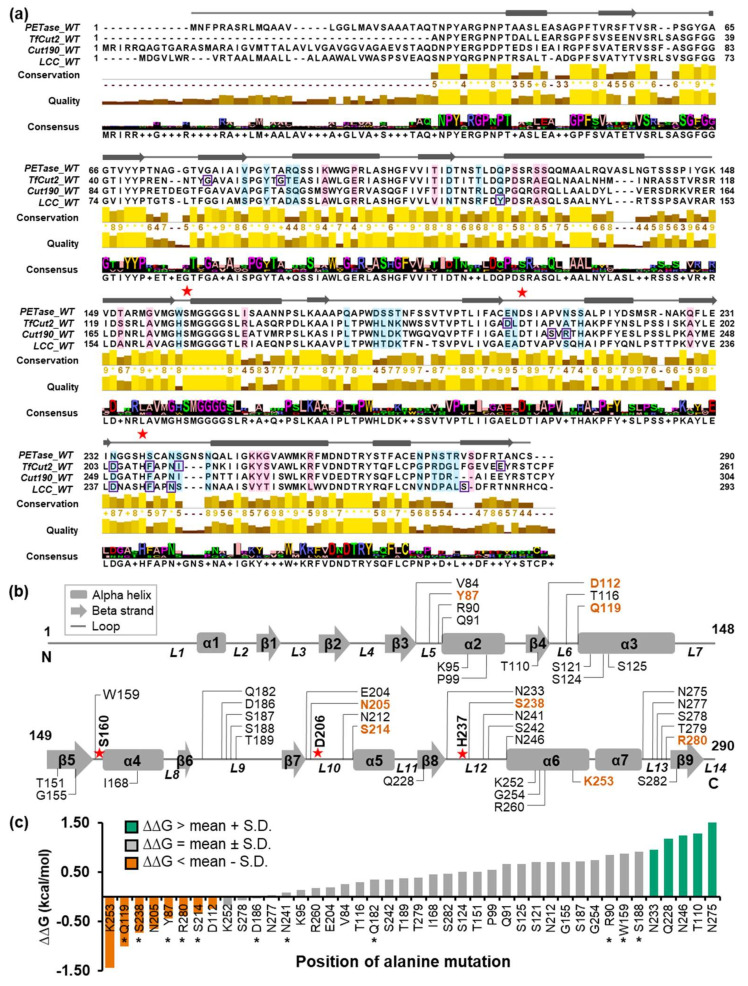
MSA of PET-degrading enzymes and the free binding energy of the PETase-PET complex. (**a**) Amino acid sequences of four PET-degrading enzymes were aligned. The PETase secondary structure elements were indicated in the top panel with a gray rectangle and arrow for α-helix and β-sheet, respectively. Amino acid sequences with low conservation scores were highlighted with blue and pink boxes for the loop and the non-loop region residues. Reported amino acid sites for mutagenesis were in purple boxes. The conservation, quality, and consensus were illustrated at the bottom. (**b**) The positions of 42 selected amino-acid residues of PETase were indicated in the secondary structure. (**c**) The bar graph showed the relation between the ∆∆G of each PETase variant-PET complex and the position of alanine mutation. The conformation of the PET substrate (2-HE(MHET)_4_) binding to PETase was selected to determine ∆G, which was then compared with that of the WT PETase, ∆∆G. The asterisks specify the validated residue in the previous studies, Figure S7. (**a**,**b**) The S–D–H catalytic triad was marked as red five-pointed stars. (**b**,**c**) The eight selected amino acid residues were highlighted in orange.

PETase amino acid sequences were compared to those of well-studied homologous enzymes that naturally and effectively hydrolyze PET under optimal conditions, such as TfCut2, Cut190, LCC, and their improved variants, in order to identify a potential PETase mutation site [14,15,18,41]. MSA was used to predict amino acid sites that affect PET-degrading activity (Figure 1a). The amino acid sequences of these PET-degrading enzymes were then aligned to determine their sequence–structure–function relationships (Figure 1a). PETase amino acid sequences matched with TfCut2, Cut190, and LCC by 44.93%, 40.51%, and 43.71%, respectively. Although each enzyme originated from different organisms, the catalytic triad of PETase consists of a serine (S160), an aspartate (D206), and a histidine (H237). The S–D–H residues act as an electron transfer route, with S160 donating electrons to the substrate. We looked for potential mutation sites in the PETase–PET interaction by observing residues near the active site. There were 42 sites chosen in total, consisting of 27 loop-region residues and 15 non-loop-region residues (Figure 1b and Figure S1a). These residues were chosen on the assumption that amino acid sites with lower conservation scores among homologous proteins are not evolutionary conserved and are more likely to evolve quickly; thus, mutating these sites could improve protein–substrate binding [42,43].

**Figure 2:**
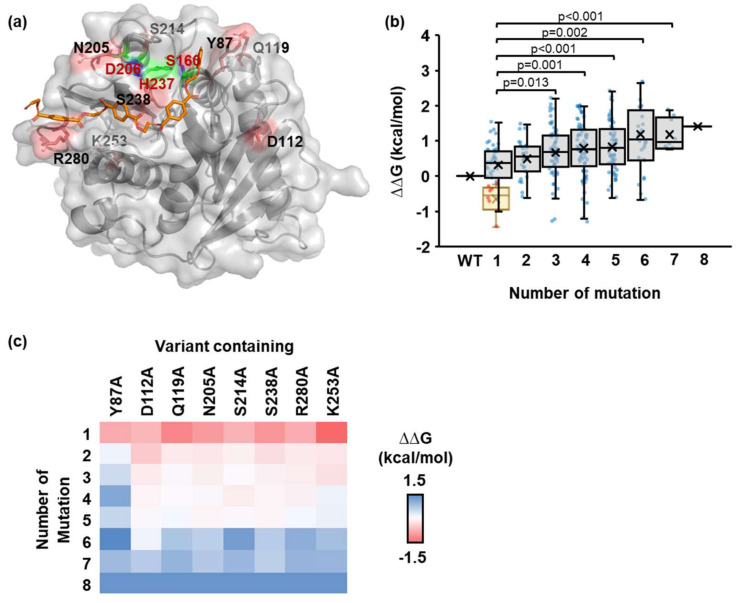
∆∆G of PETase variants using the iterative *in silico* alanine scanning mutagenesis. (**a**) The surface presentation of PETase (PDB ID: 5XJH). The catalytic triad, PET substrate docking model, and selected residues were highlighted in green, orange, and red, respectively. (**b**) The boxplot showed the ∆∆G distribution of the PETase variant and PET substrate complex with respect to the WT PETase. Each variant contains a different number of mutation sites, ranging from one to eight sites. The blue dots presented the ∆∆G of an individual PETase variant. The yellow box represented the eight selected variants with ∆∆G below the threshold. (Unpaired two-tailed Student’s *t*-test, p values were indicated in the graph). (**c**) The color map illustrated the ∆∆G distribution as a function of the number of mutation sites and the presence of the fixed amino acid that appears in PETase variants.

The individual site was then alanine substituted, yielding 42 PETase variants for molecular docking screening. The docking conformation with the PET substrate fitting in the active site of each PETase variant that provided the lowest ∆G was chosen for further analysis (Figure S1b). The findings revealed that 11 of the 42 variants with predicted mutation sites on the loop region (Y87A, D112A, Q119A, D186A, N205A, S214A, S238A, S278A, and R280A) and the non-loop region (K252A and K253A) had lower ∆G than the wild-type (WT) PETase (Figure 1c). PETase variants with negative ∆∆G mutated residues could be used as potential mutation sites for increasing the affinity of the PETase–PET interaction [44,45,46]. Only eight mutation sites were nominated for iterative *in silico* ASM, including Y87, D112, Q119, N205, S214, S238, R280, and K253 (Figure 2a), with ∆∆G below the threshold energy defined as ∆∆G—S.D., which were nominated for iterative *in silico* ASM (Figure S2a).

### 2.2. Identification of the Number and Position of Mutation Sites

A total of 255 PETase variants were created by combining eight different mutation sites. Each variant was subjected to molecular docking simulation with the PET substrate, and the individual ∆∆G was calculated with respect to the WT for each docking conformation. PETase variants with one or two mutation sites had the lowest overall ∆∆G value, according to our findings (Figure 2b,c). Increasing the number of mutation sites beyond two decreased the likelihood of finding the desired variants (Figure S2b), indicating that up to two PETase mutation sites are sufficient for mutagenesis, despite the fact that a few variants containing three to six mutation sites also showed negative ∆∆G (Figure 2b and Table S1).

The specific sites that have a significant impact on the docking score differ depending on the number of mutation sites. We discovered that K253A variants had the lowest ∆∆G for a single mutation site (Figure 2c). In the case of two-mutation-site variants, a group of variants containing either D112A or S238A had the lowest overall ∆∆G (Figure 2c and Figure S2c). These findings suggested that combining multiple low ∆∆G single-mutation sites does not always yield the desired low ∆∆G variants. Here, the variants with an alanine substitution at position 112 appeared to have the lowest ∆∆G, while variants with an alanine substitution at position 87 appeared to have the highest ∆∆G (Figure 2c).

### 2.3. Identification of Physicochemical Properties of Side Chains of PETase

**Figure 3:**
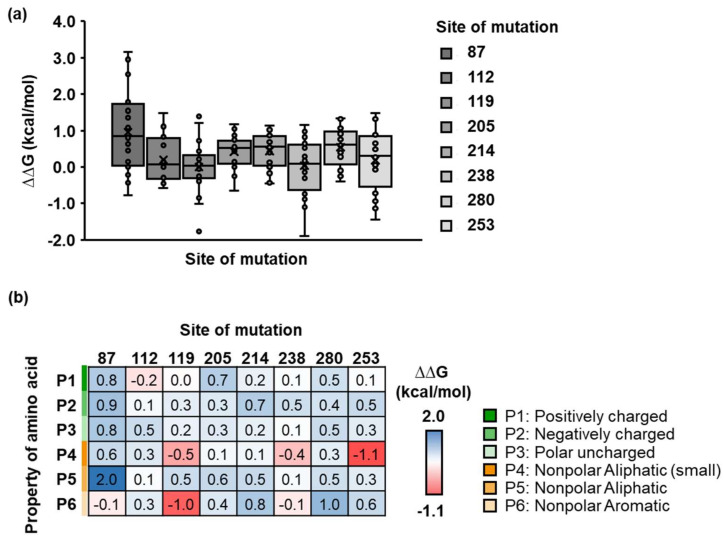
Single site-saturation mutagenesis of PETase on a selected mutation site. (**a**) The boxplot showed the ∆∆G distribution of the PETase variant and PET substrate complex with respect to the WT PETase. The dots showed the ∆∆G of an individual PETase variant of each mutation site. (**b**) The color map illustrated the ∆∆G distribution as a function of the mutation sites and the physicochemical properties of side chains (P1–P6 described in the legend).

Based on the results of *in silico* ASM, PETase variants with one to two selected mutation sites is preferable for improved PETase binding due to a higher chance of finding the desired variants. The selected amino acid sites were chosen to characterize the side chain properties via computational SSM. Eight selected sites were subjected to random mutagenesis for a single mutation site, and each variant was tested using molecular docking against the PET substrate (Figure S3). The variants with mutation sites at positions 119 and 238 had the lowest overall ∆∆G (Figure 3a), and the physicochemical side chain properties on each mutation site affected the binding free energy differently (Figure 3b). For example, substituting polar uncharged (Gln) for the nonpolar aromatic side chain (Phe) at position 119 (Q119F) or substituting the hydroxyl group (Ser) for the thiol group (Cys) of the polar uncharged side chain at position 238 reduced the docking energy (Figure S3). As a result, the Q119F or S238C variant could be a viable candidate for PETase binding improvement.

**Figure 4:**
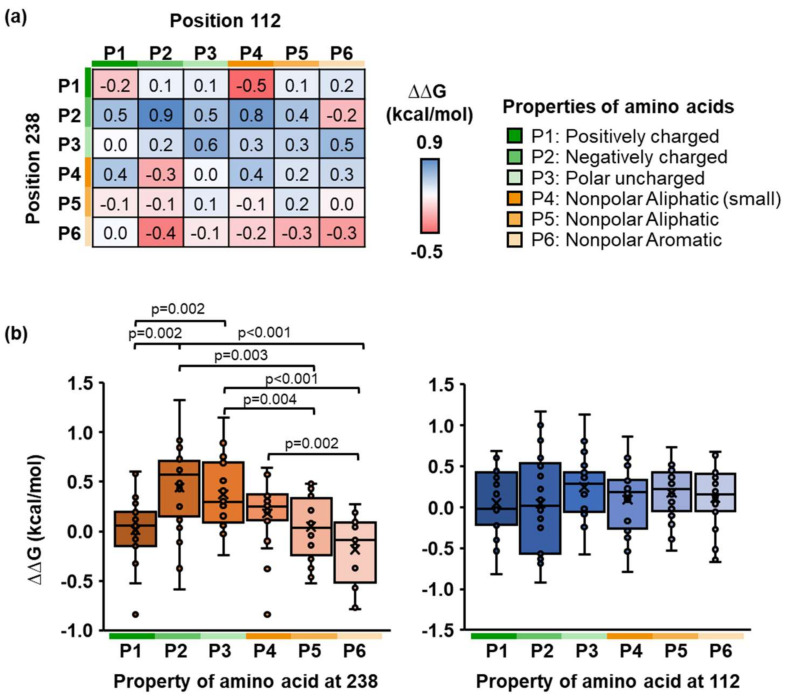
Double site-saturation mutagenesis of PETase on positions 112 and 238. (**a**) The color map illustrated the ∆∆G distribution as a function of the physicochemical properties of side chains (P1–P6 described in the legend) at positions 112 and 238 of PETase. (**b**) The boxplot showed the ∆∆G distribution of the PETase variant and PET substrate complex with respect to the WT PETase. An individual box represented different physicochemical properties of amino acids (P1–P6) at position 238 (left) and position 112 (right). The dots presented the ∆∆G of an individual PETase variant containing random mutagenesis at position 112 (left) and 238 (right). (Unpaired two-tailed Student’s *t*-test. Left: *p* values are indicated in the graph. Right: No significant difference at *p* < 0.05).

For a double mutation, the random mutagenesis was performed on positions 112 and 238. Both sites were substituted with different amino acids, resulting in a total of 400 molecular docking variants. Each variant was then shown in the protein sequence space, where the x- and y-axes represented grouped amino acids based on side-chain properties at positions 112 and 238, respectively (Figure 4a). Herein, physicochemical properties of the PETase–PET complex had a significant impact on its binding energy. For example, the variants with negatively charged (or small aliphatic) and aromatic (or positively charged) residues at positions 112 and 238, respectively, have the lowest docking score. Furthermore, the amino acid properties at position 238 are more dominant than those at position 112. (Figure 4b). We discovered that the PETase variant with the D112M/S238F mutation had the lowest ∆∆G among 400 variants in a single individual (Figure S4).

### 2.4. Substrate Binding Visualizations from MD Trajectories of PETase and Variants

**Figure 5:**
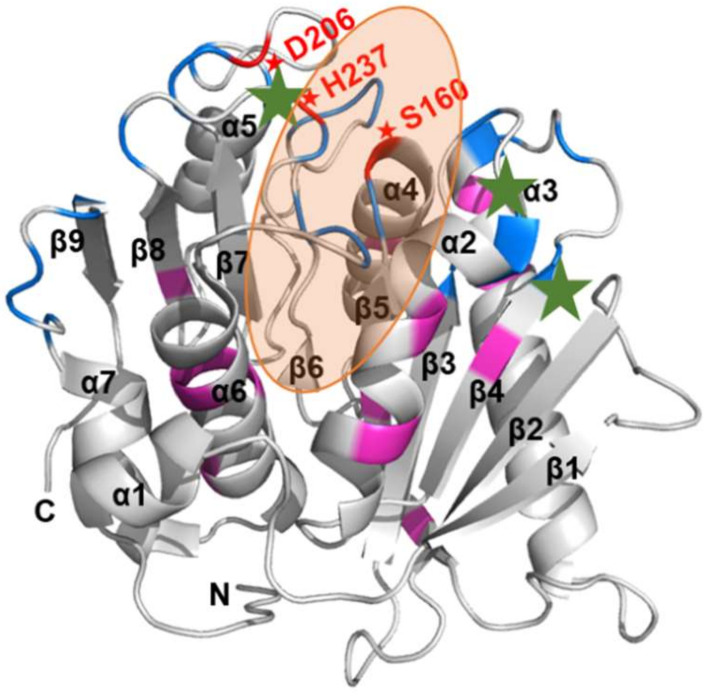
Three-dimensional ribbon representation of the WT PETase. A PETase enzyme displayed the nomenclature for N-terminus (N), C-terminus (C), all alpha helices (α1–α7), and all beta strands (β1–β9). Catalytic residues (S160, D206, and H237), and the selected mutated residues 112, 119, and 238, were denoted by red and green stars, respectively. The orange shadow represented the binding pocket. Amino acid sequences in the loop or non-loop regions were highlighted with blue or pink (related to Figure 1a).

A series of atomistic MD simulations were performed to investigate the structures and dynamics of the selected PETase variants and PET complexes in the physicochemical environment to validate the molecular docking results. According to our molecular docking results and previous researches [22,23,24], the PETase binding pocket, represented by the orange ellipse in Figure 5, spanned some parts of the helices α2–α6 and strands β5–β7. In addition, the peptide loop β3/α2, the β7/α5 loop containing an active residue D206, and the β8/α6 loop containing another active residue H237 were discovered to interact with the substrate.

Given the geometry of the binding pocket (Figure 5), simultaneous binding of the substrate on β3/α2, β7/α5, and β8/α6 loops allowed the substrate to interact with the active residue S160 buried inside the binding pocket. Mutated sites from the docking results (green stars in Figure 5) form a triangle around the binding pocket. As mentioned, in comparison to the WT PETase, three complexes with the lowest docking score predicted by molecular docking were chosen for MD simulations, including PETase variants with one mutation site (Q119F and S238C) and two mutation sites (D112M/S238F). Even though some mutation sites, such as S238L, S238F, and K253A, gave the promising score, the tetramer substrates were bound to the active residues by their ends that could not facilitate the chain cleavage (Figure S5). Therefore, these three mutants were excluded.

**Figure 6:**
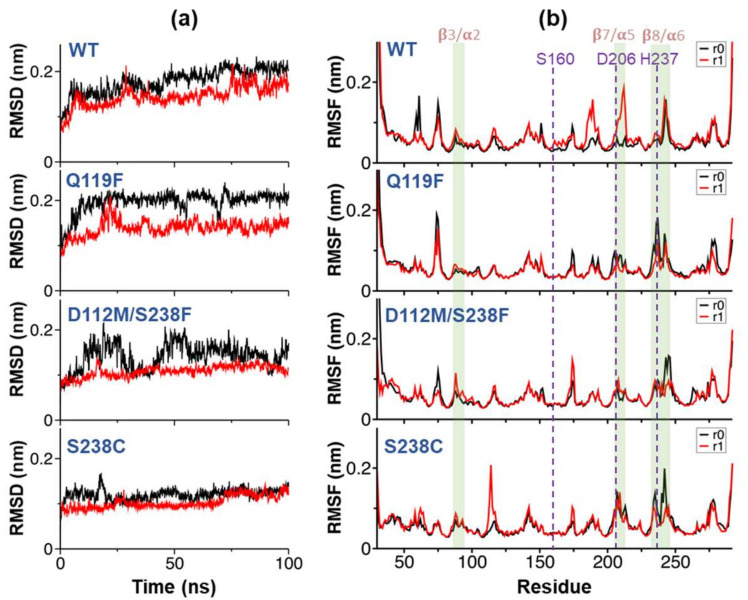
Simulations and conformational analysis. (**a**) Root-mean-square deviation (RMSD) of the proteins during the course of 100 ns NPT simulations compared with their starting structures based on the crystallographic data. The RMSD results of WT, Q119F, D112M/S238F, and S238C were indicated with two replicas “r0” (black line) and “r1” (red line). (**b**) Per-residue root-mean-square-fluctuation (RMSF) calculated from the last 50 ns of all trajectories. The RMSF results of WT, Q119F, D112M/S238F, and S238C were indicated with two replicas “r0” (black line) and “r1” (red line). The green shadows represented regions β3/α2, β7/α5, and β8/α6 of PETase.

**Figure 7:**
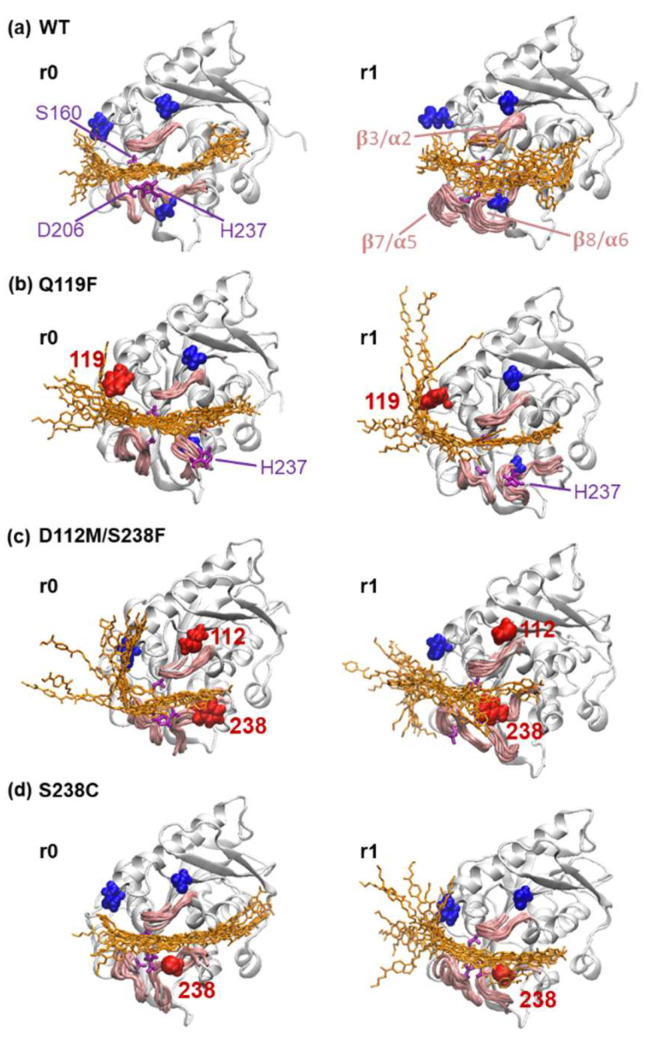
Conformational snapshots of the PETase and PET complex. Three-dimensional ribbon representation of the (**a**) WT PETase and its three mutants, (**b**) Q119F, (**c**) D112M/S238F, and (**d**) S238C, along with the superimposed snapshots taken between 50–100 ns of the tetra-PET substrates (orange), unmutated residues 112/119/237 (blue), and mutated residues 112/119/238 (red). Other important residues and regions were labeled within this figure. Two replicas “r0” (left) and “r1” (right) from each protein structure were presented.

Following the simulations, a root-mean-square deviation (RMSD) was calculated to quantify global conformational changes of PETase variants with respect to the energy-minimized starting structure (Figure 6a), along all eight MD trajectories (two replicas “r0” and “r1” from each protein structure). Despite a few minor fluctuations, the convergence of RMSD was observed in the last 50 ns of all simulations, indicating that equilibriums were reached. Per-residue root-mean-square fluctuations (RMSF) was calculated from all eight simulations (Figure 6b). The RMSF profiles roughly represented the structural characteristics of all PETase variants. The local RMSF peaks indicated the regions of flexible loops connecting secondary structures, particularly the β3/α2, β7/α5, and β8/α6 loops near active sites. Interestingly, additional RMSF peaks were observed in both MD simulation replicas of each variant near the H237 catalytic residue. The effects of point mutations on substrate-binding mechanisms and the local contribution of other amino acid residues were shown in Figure 7.

During the 50–100 ns period, MD trajectories of the WT PETase and three predicted mutants were visualized by superimposed snapshots of the tetra-PET substrate, along with the three peptide loops β3/α2, β7/α5, and β8/α6 around the binding pocket. While the WT-r0 replica showed that the substrate remained intact with three catalytic residues, the binding posture was uncertain at both ends of the substrate, and the substrate molecule and three peptide loops became more unstable in the WT-r1 replica. The interaction network, however, did not include the three key mutation residues, D112, Q119, and S238. According to Figure 7b, the addition of a hydrophobic group within the Q119F variant drew one end of the tetramer towards the mutated site F119, while the other end became detached from the catalytic residue H237.

Meanwhile, the D112M/S238F double mutant showed a significant change in substrate behavior (Figure 7c), as the D112M mutation removed the salt bridge formed between the negatively charged D112 and the positively charged R90 at the end of the 3/2 loop, and the S238F mutation increased the hydrophobicity near the catalytic residue H237. As a result, the tetra-PET substrates in both the D112M/S238F-r0 and D112M/S238F-r1 replicas became detached from the β3/α2 loop and the catalytic site S160, but remained strongly attached to the β8/α6 loop via the hydrophobic F238 residue. The mutation also increased the hydrophobicity of the S238 residue, and the mutated C238 became an additional binding site without disrupting the binding network, allowing substrate binding at the catalytic triad S160/D206/H237 to become stable for both S238C-r0 and S238C-r1 replicas.

### 2.5. MM/PBSA Analysis on the Local Contribution of Amino Acids

**Figure 8:**
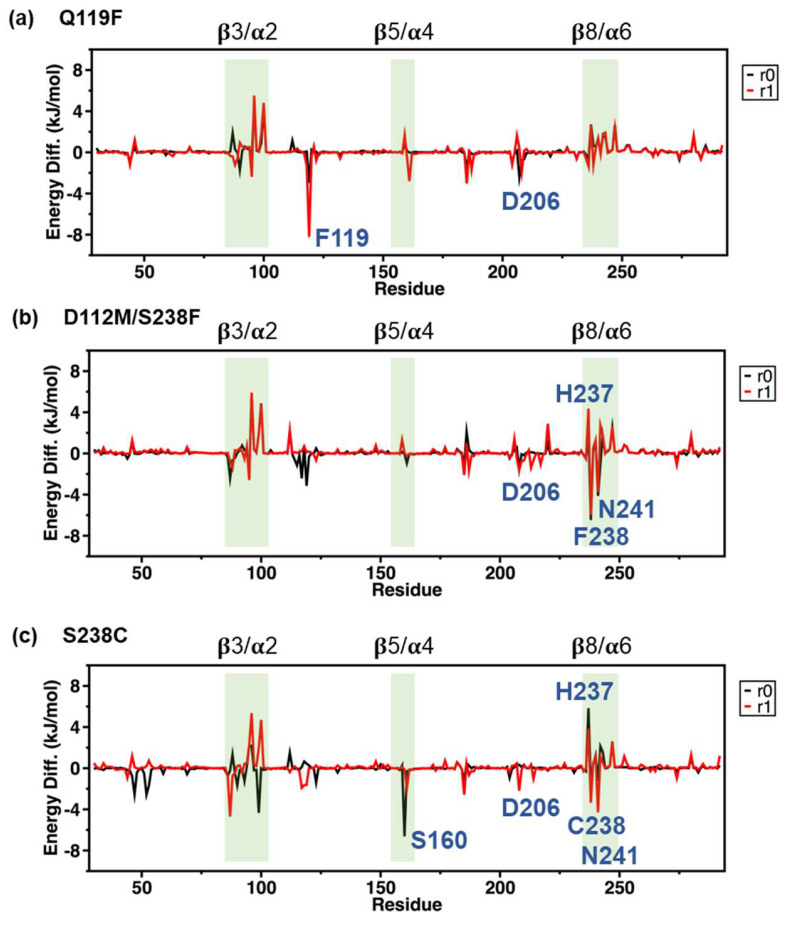
MM/PBSA analysis. The differences of per-residue MM/PBSA binding free energy decomposition of (**a**) Q119F, (**b**) D112M/S238F, and (**c**) S238C complexes relative to the WT complex were shown in two replicas (“r0”—black line and “r1”—red line). Important peaks at regions β3/α2, β7/α5, and β8/α6 were highlighted in green.

To investigate the key residues of PETase variants such as Q119F, S238C, and D112M/S238F in forming the complex with the PET substrate, the binding free energy contributed from van der Waals interactions, electrostatic interactions, polar solvation, and non-polar solvation was calculated using the MM/PBSA method on the last 50 ns of all MD simulations [47,48,49,50]. Table 1 showed the effect of each interaction energy term on each PETase variant. Only the S238C variant had a greater (S238C-r0 replica) or equal (S238C-r1 replica) MM/PBSA binding energy to the substrate comparing to the WT. The van der Waals term played a significant role in the binding energy loss caused by the Q119F and D112M/S238F mutations, which coincided with the loss of van der Waals contacts when the substrate drifted out of the binding pocket. The per-residue binding energy gain decomposition (Figure 8) showed the difference between the per-residue binding energy profile ∆∆G of each variant and the average binding energy profile of two WT replicas. Thus, negative values in Figure 8a–c represented the binding energy gain contributed by each amino acid, while positive values represented the binding energy loss.

Peaks of MM/PBSA binding energy gain at F119 residue were observed in Figure 8a for both Q119F-r0 and Q119F-r1 replicas, which corresponded to the previously mentioned extra hydrophobicity. A small binding energy gain was observed near the D206 active residue, but a relatively large binding energy loss within the β3/α2 and β8/α6 loops contributed to a weaker overall binding free energy than WT. The hydrophobic substitution S238F resulted in a significant energy gain at F238 and N241, but binding energy loss at the catalytic site H237, indicating a shift of binding positions away from the catalytic sites for the D112M/S238F variant in Figure 8b. Binding energy loss within the β3/α2 loop was also observed for the D112M/S238F variant, which contributed to a lower overall binding free energy than the WT. The S238C variant’s MM/PBSA binding energy gain profile in Figure 8c showed a unique binding energy gain peak at catalytic site S160, as well as other binding energy gain/loss peaks with similar characteristics to other variants.

**Figure 9:**
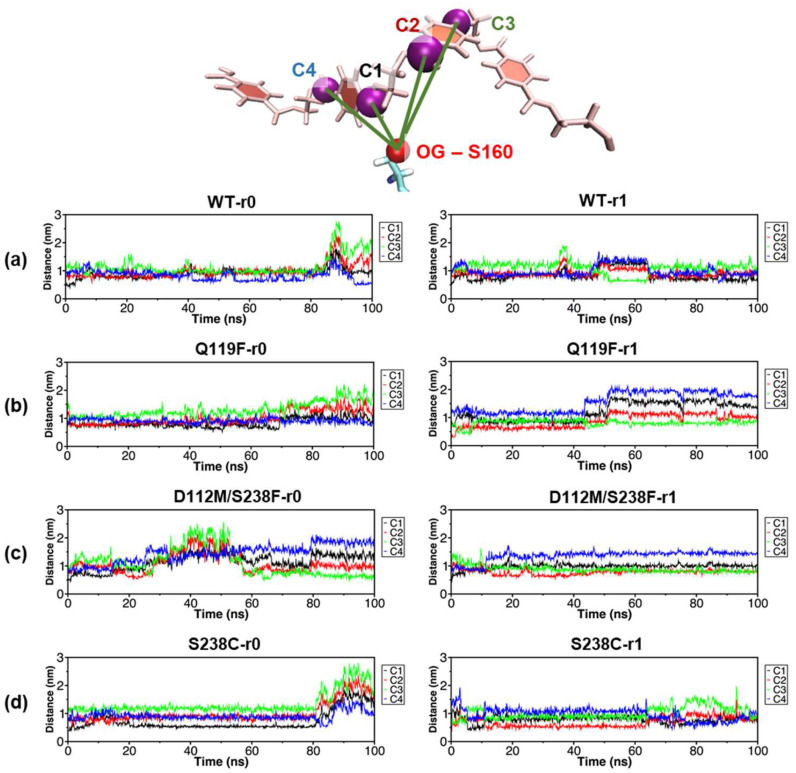
Distance between the catalytic residue S160 and the PET substrate. The distance between the side chain oxygen atom (OG) of the catalytic residue S160 and four carbonyl carbon atoms (C1, C2, C3, and C4) within a PET tetramer was measured from both simulation replicas (r0 and r1) of (**a**) WT, (**b**) Q119F, (**c**) D112M/S238F, and (**d**) S238C.

The S160 amino acid residue was important in the hydrolysis of PET substrates because it transferred an electron from its side chain hydroxyl oxygen atom (OG) to a carbon atom within a PET carbonyl (C=O) group [23]. As a result, throughout the simulations, the distances between the OG of S160 and four carbon atoms from four middle carbonyl groups of the modelled tetra-PET substrate were measured (Figure 9a–d and Figure S6). The distances between OG and the two center-most carbon atoms (C1 and C2) of the carbonyl groups were minimal at the start of each simulation, which corresponded to the docking results. The substrate slid along the binding cleft when either C3 or C4 became closest to the OG of S160. Substrate sliding was observed in the Q119F-r1 and D112M/S238F-r0 simulations where C4 was moved further away from the S160 residue. Meanwhile, in both S238C simulations with an additional contribution of S160 to the MM/PBSA binding energy, C1 and C2 remained the closest atoms to the OG atom of S160, possibly facilitating electron transfer.

### 2.6. Local Effects Induced by the S238C Mutation on the PET Substrate Binding

**Figure 10:**
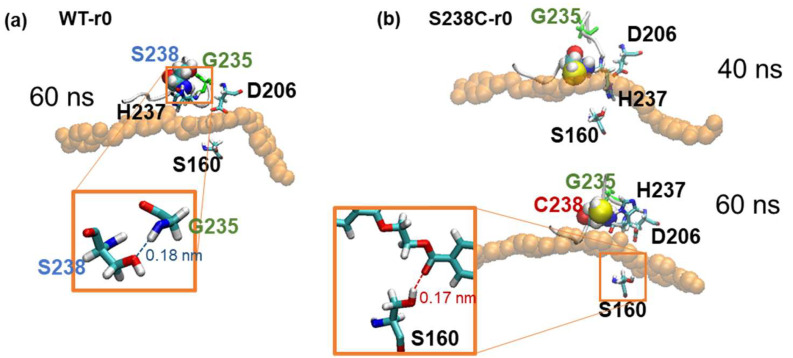
Effect of mutations on the binding pocket. (**a**) A sample snapshot taken from the WT-r0 MD simulation after 60 ns, (**b**) sample snapshots taken from the S238C-r0 MD simulation after 40 ns (top) and 60 ns (bottom). The PET tetramer was represented in orange. All three catalytic residues (S160, D206, and H237) were highlighted along a glycine residue 235 and the mutated residue 238.

Selected conformational snapshots from the WT-r0 and S238C-r0 were shown in Figure 10a,b to further investigate the effects of the S238C mutation, which caused binding pocket narrowing and an extra hydrogen bonding at S160. Snapshots of catalytic residues, mutated residues, substrate, and the residue G235 were chosen from 40 ns and 60 ns before and after the binding mode transition of the S238C-r0 replica, respectively. The native S238 residue formed a hydrogen bond with the backbone of G235 in the WT complex (Figure 10a). When the residue 238 in Figure 10b was mutated into C238, the mutated residue 238 lost its hydrogen bond with G235 and became bound to the substrate instead. The S238C mutation involved only changing an O atom into an S atom, which decreased the polarity of the side chain and increased van der Waals contacts with the substrate due to a larger van der Waals radius of an S atom than that of an O atom.

The additional van der Waals contacts formed between C238 and the substrate, as well as the hydrogen bond loss, were consistent with the RMSF profile chain fluctuation around C238 and the narrowed binding pocket confirmed by the reduced distances between catalytic residues. After 60 ns, H237 was seen drifting away from another catalytic residue S160, but the substrate moved in close proximity to S160, and a hydrogen bond was formed between the side chain of S160 and the substrate’s carbonyl group. This bimodality of substrate binding with the S238C variant demonstrated the potential conformational changes during a multi-step electron transfer facilitated by the additional hydrophobic contacts with the substrate and the increased flexibility of the binding pocket.

## 3. Discussion

Despite extensive investigation of the PETase active site via crystallography studies, molecular modeling [22,23,26], and sequence-structure analysis of PETase with other known PET-hydrolyzing enzymes [51,52], the actual binding mode of PET to PETase is still under investigation. The PET tetramer was docked to the PETase to identify potential mutation sites. MD simulations were then run to investigate the molecular interaction between them. Our observed binding mode was consistent with previous reports [22,23]. The solid-state NMR indicated that the PET substrate is highly stiff, leading to the binding of all four monomers onto the previous proposed binding cleft of PETase, which is unlikely [53]. This suggested that the PET dimer may be more suitable for computational modeling. However, alanine substitutions on Y87 and R280 located at the beginning and end of the L-shaped binding cleft had an effect on PETase activity [23,24,25,35]. Therefore, at least four monomers of the PET substrate (or six monomers [35]) are reasonable representations of the PET polymer in the computational modeling scheme to cover the binding cleft.

To improve PET-degrading performance, *in silico* alanine scanning and site-saturation mutagenesis were used to look for potential PETase mutation sites. Molecular docking was chosen to screen PETase variants from the large protein space because it is computationally efficient. MD simulations elucidated the conformational changes of perturbed protein structures toward a new equilibrium that could either facilitate or hinder substrate binding. Differences in binding free energy (docking score) between the protein–substrate complex and the WT are commonly used as indicators to determine binding affinity and the significance of a specific amino acid residue because the efficiency of PET-degrading activity is related to the binding affinity of PETase and the PET substrate [23,38,44,45,46]. Furthermore, the chemical steps involved in the depolymerization of PET have an impact on the degradation efficiency. Five (62.5%) of the eight identified mutation sites from our screening were in good agreement with prior experimental studies (Figure S7) [23,24,25,31], while the remaining (37.5%) had not previously been reported. As a result, the dependability of the screening protocol we presented is validated. Despite the fact that molecular docking and MD simulations revealed positions 57–64 (the β1–β2 connecting loop (L3), Figure 1) to be flexible and thus suitable for mutagenesis, no experimental results were reported [30].

One of the most important factors influencing protein structure and function is the number of mutation sites. Several improved PETase mutation sites have been investigated (Figure S7), including one [23,24,31], two [22,32], three [32], and ten [54]. Even though one and two mutation sites were the most commonly reported, the degrading activities of those variants cannot be directly compared due to differences in experimental conditions (e.g., lysis buffer, time, concentration, and target substrate—PET film or bottle). Up to two mutation sites could be preferable for PETase activity improvement via the SSM approach [35]. Although we discovered that a few PETase variants with more than two mutation sites have stable docking conformations [32,54], the probability of finding these beneficial variants was low due to the difficulty in computationally and experimentally searching in a high-dimensional protein sequence space. Alternatively, structure-based, machine learning was recently used to predict potential mutations of PETase [55]. In addition to the number of mutation sites, increasing PETase thermal stability is another factor to consider for activity, as demonstrated by the successful example of PETase with three mutated residues that stabilized the β6–β7 connecting loop [32].

Exploring the PETase sequence space can help identify PETase variants with higher PET-degrading activity. The interaction between the PETase and PET substrate was highly involved in variants containing a single mutation site, either on position 119 or 238, which was consistent with the previous report [22]. Similar to the Q119Y mutation, an aromatic residue at position 119 (Q119F) could improve hydrophobic packing in the binding pocket of the PETase structure and form interactions with the aromatic motif of the PET substrate [54]. This increase in hydrophobicity at one end of the binding pocket may strengthen substrate binding while unbinding the substrate from the other end, which may only be the case for a short substrate. At position 238, changing the polar-uncharged Ser (an O atom) to Cys (an S atom) can improve the PETase–PET complex interaction. The S238C resulted in a hydrogen bond loss within the peptide loop containing a catalytic residue, which was compensated by the additional hydrophobic contact between the substrate and the more hydrophobic S atom.

Many studies have chosen position 238 as a mutation site because it is close to one of the catalytic triads (H237). Replacing 238 with a smaller side chain, such as Ser or Arg, instead of a conserved Phe, increases the flexibility of the active site and improves the interaction between PETase variant and PET [23,26]. However, double mutations of the W159H/S238F PETase variant also improved PET-degrading performance because these mutations changed the space of the binding cleft, which led to a shorter distance between S160 and the substrate [22]. Given the properties of the side chains and the distance between S160 and the substrate, our S238C variant may improve flexibility while narrowing the binding pocket that brings the substrate close to S160. Our protein sequence space revealed a variety of side chain properties at different mutation sites at positions 87, 214, and 280, which were consistent with experimental findings [23,24].

Although 62.5% of our predicted PETase variants with a single mutation site agreed with experimental studies, no report on the PETase variant with two mutation sites at positions 112 and 238 was found. As a result, we used MD simulations to investigate the interaction of the D112M/S238F variant with PET. Interestingly, while the S238F mutant was reported as a positive mutation candidate for double mutation, W159H/S238F [22], position 112 at the C-terminus of β4 had not been chosen for any double mutation study. Molecular docking predicted that D112M would add hydrophobic contacts to the substrate. However, MD simulations revealed that D112M destabilized the binding pocket by causing the salt bridge to fail. The discrepancy between docking and MD results was caused by protein dynamics, which were typically ignored in molecular docking.

Molecular docking remained an ideal calculation technique for rapidly screening potential candidate amino acids for *in silico* ASM and SSM. However, the predicted mutants’ binding affinity and PET-degrading activities must be validated further using MD simulations and enzymatic activity assays to account for any detrimental effects on the overall structure of the proteins. We believe that our research will provide beneficial information about the number, position, and side chain properties of PETase mutation sites that can be used to improve the enzyme’s PET-degrading performance. Still, more mutations would be encouraged for the MD simulation to help understand other PETase variants and consolidate the simulation protocol. Furthermore, not limited to PETase, our protocol could act as a preliminary guide to identify potential mutation sites of other PET-degrading enzymes, such as BbPETase from bacterium *Burkholderiales* [56], and MoPE from bacterium *Moraxella* sp. [57].

## 4. Materials and Methods

### 4.1. Multiple Sequence Alignment

The following FASTA sequences of homologous PET-degrading enzymes were retrieved from the Protein Data Bank: WT PETase (PDB ID: 5XJH), TfCut2 (PDB ID: 4CG1), Cut190 (PDB ID: 4WFJ), and LCC (PDB ID: 4EB0). Furthermore, the TfCut2 variants G62A/F209A, G62A/I213S, and D204C/E253C/D174R [14,15]; Cut190 variants S226P/R228S [41]; and LCC variants, such as F243I/D238C/S283C/Y127G, were selected as the PETase candidate amino acid sites. Jalview [36] and the Clustal Omega web service [37] were used to investigate the sequence relationships among the enzymes of interest, as well as the conservation scores, which are based on the similarity of physicochemical properties for the amino acid in each column, of specific residues and positions (default parameter settings).

The candidate was chosen based on amino acid sites in the loop region on the same side as the active site, or those with a low conservation score in the non-loop region (i.e., alpha helix or beta sheet). In general, amino acid sites with conservation scores less than 6 were chosen. However, if the mutation sites were reported by other studies, some of the amino acids with high conservation scores were also included for method validation [22,23,24,25,31,32]. In total, 42 sites were chosen, with 27 amino acids in the loop region and 15 amino acids in the non-loop region.

### 4.2. Preparation of Enzyme Structure for Molecular Docking

PETase crystal structure was obtained from the RCSB PDB (5XJH) [23]. Individual PETase variant was created by using the mutagenesis tool in PyMOL (https://pymol.org/2/, accessed on 17 March 2022) [58] to replace 42 selected amino acid sites with alanine, yielding 42 PETase variants. To simulate the condition at pH 7, the important titratable Asp residues were deprotonated for each variant. The PET tetramer structure of four repeat units of MHET (2-hydroxyethyl-(monohydroxyethyl terephthalate)_4_) or 2-HE(MHET)_4_, capped at both ends with an ethanol group, was built and optimized for the ligand using Argus Lab (http://www.arguslab.com/, accessed on 17 March 2022).

### 4.3. Molecular Docking

PETase’s crystal structure was used as a macromolecule, and the ligand and water molecules were removed. The polar hydrogens were then added to the protein in its default orientation, and AutoDock4 calculated the protein’s Gasteiger charges. The hydration state of the substrate was treated using the method described previously [59,60]. All other docking parameters for the proteins and substrate were from Auto-default Dock’s settings, with a grid box size of 120 × 90 × 120 Å^3^ covering the active site. The grid box was centered at the coordinates x: −2.403, y: 30.711, and z: −18.311. For the energetic map calculations, a grid spacing of 0.375 Å and a distance-dependent function of the dielectric constant were used. The Lamarckian genetic algorithm was used to find the native-like binding conformation, with a population size of 200; a generation limit of 27,000; and 2,500,000 energy evaluations.

The 500 independent docking iterations were performed using Autodock4.2 [39,59], and their binding conformations were obtained to create a heatmap of the substrate’s binding energy in the active site of PETase. The docking results were then clustered using a RMSD tolerance of 2.0 Å, which was applied to all docking calculations. The docking pose of the PET tetramer in each PETase structure was chosen if the distance between the PET substrate and the OH of S160 was less than 4 Å, and the PET substrate conformation lay along an L-shaped cleft (subsite I and II as shown in Figure S1b) covering the active site [23]. For further analysis, only the lowest binding free energy (docking score), defined as ∆G, from the same docking pose of each PETase–PET complex, was chosen.

### 4.4. Candidate Mutation Selection

The ∆G of each variant in the complex with the PET substrate was measured using AutoDock and was calculated based on the empirical free energy force field, which is the sum of the intermolecular energies (e.g., dispersion/repulsion, electrostatics, hydrogen bonding, and desolvation) and the torsional free-energy penalty from the formation of the complex between the protein (PETase variant) and the ligand (PET substrate). The difference between the docking score of each variant (∆G^V^) and the WT (∆G^WT^), defined as ∆∆G = ∆G^V^ − ∆G^WT^, was calculated and ranked based on the ∆∆G. The lower value of the ∆G indicates the higher energetically favorable binding conformation. Later, the selection threshold criteria were computed (the mean value minus the standard deviation (S.D.) of all ∆∆G from 42 variants). As a result, eight candidate mutation sites with ∆∆G below the threshold were selected for iterative ASM.

### 4.5. Alanine Scanning Mutagenesis

Multiple rounds of ASM with different permutated alanine substitution positions to identify the number and position of mutation sites were varied up to eight alanine mutation sites. The total number of PETase variants was calculated from the combination theory $C_{r}^{n}=n!/\left(\left(n-r\right)!r!\right)$, where *n* is the total number of objects in the set (i.e., total selected sites of amino acids. Here, *n* equals to 8.), and *r* is the number of objects chosen from the set (i.e., the number of mutation sites that ranges from one to eight sites). As a result, the total number of PETase variants with one to eight mutation sites was 8, 28, 56, 70, 56, 28, 8, and 1, in that order (a total of 255 variants). PyMOL’s Mutagenesis Wizard tool was used to perform the specific alanine substitution. AutoDock4 optimized the hydrogen atoms and charges of PETase variants. The same protocol was used to generate all 255 variants.

### 4.6. In Silico Site-Saturation Mutagenesis

Eight PETase mutation sites were individually subjected to random mutagenesis using PyMOL’s mutagenesis tool, with all residues in their standard pKa during the simulation. Local optimization was performed for each mutated site by selecting the most likely conformation of the newly substituted amino acid. Each selected mutation site was substituted to other amino acids for single-site saturation mutagenesis, resulting in 19 PETase variants for each mutation site. As a result, there were 20 × 8 variants (including the WT). For two-site saturation mutagenesis, amino acid sites at positions 112 and 238 were chosen and randomly mutated, yielding 20 × 20 PETase variants. The PET tetramer structure of four repeat units of MHET was used as the ligand [22,23].

### 4.7. Molecular Dynamics Simulations and Analysis

A GROMOS54A7 force field [61] in GROMACS v5.1.2 [62] was applied to perform MD simulations. Semi-empirical calculations embedded within the Automated Topology Builder webserver [63] and the GROMOS54A7 force field were used to generate force field parameters for each ligand from the selected structure of each protein–ligand complex as determined by the AutoDock4 molecular docking results. For MD simulations, the following steps were taken. The center of mass for each protein–ligand complex was placed in the center of a simulation box (8 nm × 8 nm × 8 nm) with the remaining space filled with approximately 10,000 SPC water molecules [64]. In all simulations, a cut-off distance of 0.1 nm was used for short-range interactions.

Following a 50,000-step energy minimization by the steepest descent algorithm, a 1-ns simulated annealing simulation was run. Thus, in an NPT ensemble with a velocity-rescaling thermostat and a Berendsen barostat, the system was linearly heated from 100 to 300 K at 1 atm [65]. Finally, the system was subjected to 100-ns MD runs at 300 K, 1 atm, and with an NPT ensemble equipped with a velocity-rescaling thermostat and a Parrinello–Rahman barostat [66]. Only internal motions of protein–ligand complexes were studied for data analysis, regardless of water molecules and translational or rotational motion. The RMSD from each simulation was calculated every 10 ps to track the conformational changes of these complexes. Furthermore, the RMSF were calculated locally from each simulation to assess the flexibility and rigidity induced by ligand binding.

### 4.8. Binding Free Energy Analysis

The ligand binding free energy of PETase was computed using the Molecular Mechanics/Poisson Boltzmann Surface Area (MM/PBSA) method via g_mmpbsa package [47] in GROMACS. The total free energies of protein (G_protein_), ligand (G_ligand_), and protein–ligand complex (G_complex_) were categorized into MM potential energy in vacuum (E_MM_), polar solvation term (G_polar_), and non-polar solvation term (G_apolar_), which were used to calculate for the binding free energy between PETase and PET (∆G_binding_ = G_complex_ − (G_protein_ + G_ligand_)) in the solvent.

The calculation enthalpy in a vacuum was based on GROMOS 54a7 forcefield regardless of the entropic contribution. The Adaptive Poisson Boltzmann Solver routines were performed [67] to determine the implicit solvation energy, with the dielectric constants of protein and water (4 and 80, respectively). The solvent-accessible surface area (SASA) model was used to calculate the non-polar solvation energy with the surface tension of the solvent (0.0226778 kJ/(mol Å^2^)) and SASA energy constant (3.84982 kJ/mol). MM/PBSA calculations were performed on the equidistant 500 snapshots from the last 50-ns MD trajectory. Besides, per-residue energy decomposition was written as the contribution of individual amino acid residue.

## 5. Conclusions

Our work proposed a new protocol for protein design and engineering by combining *in silico* alanine scanning mutagenesis (ASM) and site-saturation mutagenesis (SSM) to identify potential PETase candidate mutations. As illustrated in the PETase sequence space, our research discovered that the number of mutation sites, positions, and amino acid side chain properties all had a significant impact on degradation activity. Besides, observing the side chain properties at different mutation sites in the PETase sequence space allows us to discover promising mutants that improve PETase function. Though quick ASM and SSM screens for mutations with favorable results are possible, the predicted variants must be validated further using MD simulations and/or enzymatic activity assays. As a result, our findings contributed to the field of protein engineering for the closed-loop plastic recycling industry.

## Figures and Tables

**Table 1:** MM/PBSA binding free energy of each complex.

PETase Variant	vdW Energy (kJ/mol)	Electrostatic Energy (kJ/mol)	Polar Solvation (kJ/mol)	Apolar Solvation (kJ/mol)	Total Binding Energy (kJ/mol)
WT-r0	−215 ± 35	−38 ± 27	121 ± 25	−25 ± 4	−156 ± 39
WT-r1	−158 ± 53	−55 ± 26	108 ± 44	−19 ± 6	−124 ± 62
Q119F-r0	−160 ± 18	−32 ± 19	94 ± 38	−20 ± 2	−118 ± 38
Q119F-r1	−155 ± 27	−31 ± 35	88 ± 19	−19 ± 3	−116 ± 51
D112M/S238F-r0	−176 ± 45	−30 ± 25	117 ± 24	−21 ± 6	−110 ± 70
D112M/S238F-r1	−159 ± 33	−32 ± 19	116 ± 42	−18 ± 3	−93 ± 46
S238C-r0	−209 ± 36	−53 ± 35	112 ± 31	−24 ± 4	−175 ± 40
S238C-r1	−173 ± 23	−39 ± 33	112 ± 42	−20 ± 3	−121 ± 37

## Data Availability

Not applicable.

## Supplementary Materials

The following supporting information can be downloaded at: https://www.mdpi.com/article/10.3390/molecules27103353/s1, Figure S1: PETase structure and the active site of PETase; Figure S2: results of iterative *in silico* alanine scanning mutagenesis; Figure S3: effect of single-site saturation mutagenesis on the binding free energy; Figure S4: effect of double-site saturation mutagenesis on the binding free energy; Figure S5: docking poses of the PETase and PET complex; Figure S6: minimum distances; Figure S7: experimental results of PET-degrading enzymes; Table S1: all PETase variants and their mutation sites.
