# Supplementary material for: In Silico Identification of Potential Sites for a Plastic-Degrading Enzyme by a Reverse Screening through the Protein Sequence Space and Molecular Dynamics Simulations
Source: Molecules. 2022 May 23;27(10):3353. doi: 10.3390/molecules27103353 (PMC9143596; doi:10.3390/molecules27103353)
Supplement: Supplementary file 1 [file molecules-27-03353-s001.zip › molecules-1728038-supplementary.pdf]

molecules
MDPI

Supplementary Material

In Silico Identification of Potential Sites for a Plastic-Degrading Enzyme by a Reverse Screening through the Protein Sequence Space and Molecular Dynamics Simulations

Krit Charupanit [1], Varomyalin Tipmanee [1], Thana Sutthibutpong [2,3] and Praopim Limsakul [4,5,*]

[1] Department of Biomedical Sciences and Biomedical Engineering, Faculty of Medicine, Prince of Songkla University, Songkhla 90110, Thailand; krit.ch@psu.ac.th (K.C.), tvaromya@medicine.psu.ac.th (V.T.)
[2] Theoretical and Computational Physics Group, Department of Physics, Faculty of Science, King Mongkut's University of Technology Thonburi (KMUTT), Bangkok 10140, Thailand; thana.sut@kmutt.ac.th
[3] Center of Excellence in Theoretical and Computational Science (TaCS-CoE), Faculty of Science, King Mongkut's University of Technology Thonburi (KMUTT), Bangkok 10140, Thailand
[4] Division of Physical Science, Faculty of Science, Prince of Songkla University, Songkhla 90110, Thailand
[5] Center of Excellence for Trace Analysis and Biosensor (TAB-CoE), Faculty of Science, Prince of Songkla University, Songkhla 90110, Thailand
* Correspondence: praopim.l@psu.ac.th

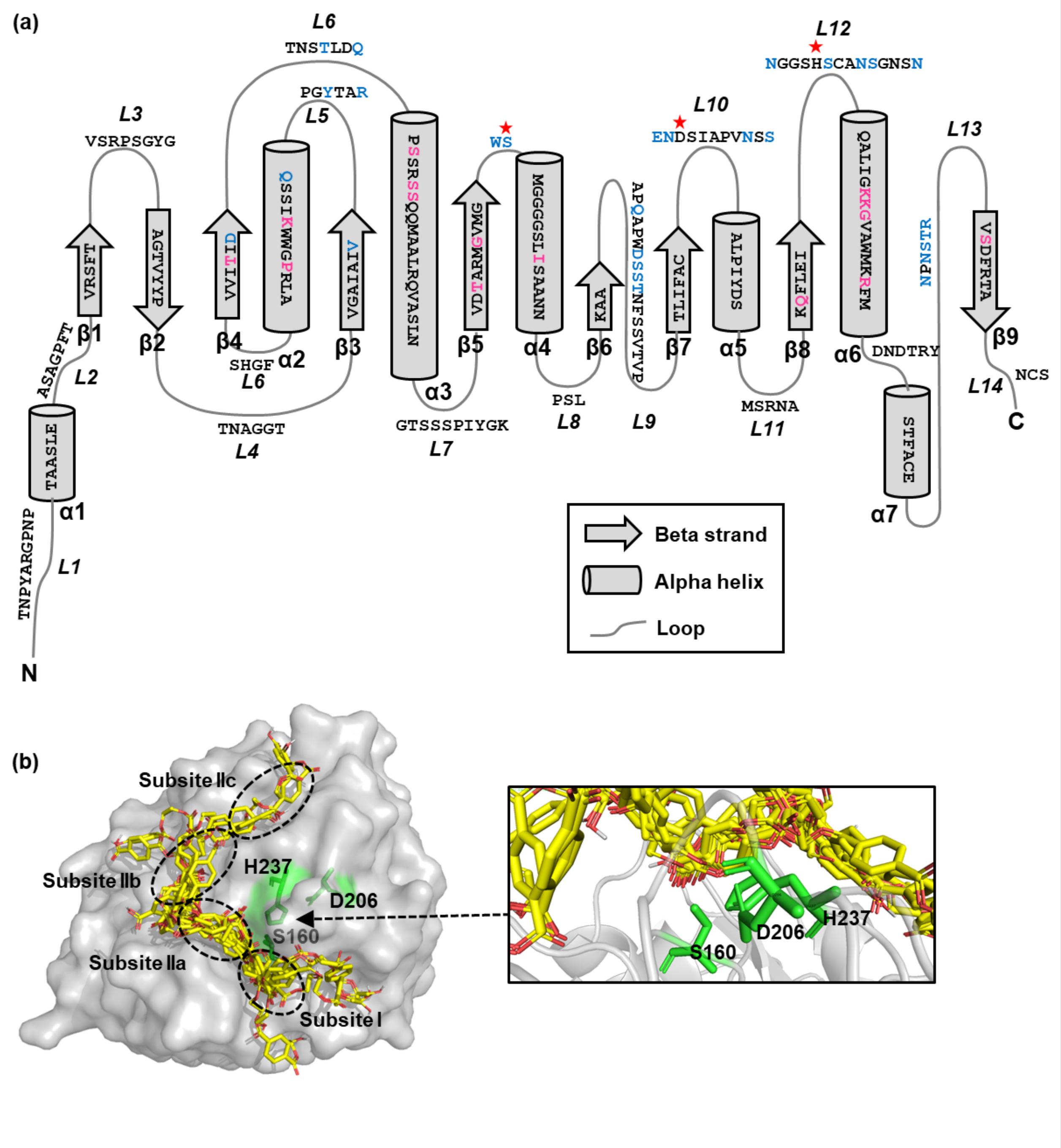

Figure S1. PETase structure and the active site of PETase. (**a**) Schematic representation of a PETase enzyme displaying the amino acid sequences on alpha helices, beta strands, and loops connecting pairs of secondary structures. Catalytic residues (S160, D206, and H237) are denoted by red stars. L represents a loop. (**b**) The docking poses of WT PETase and representative PETase variants, including Y87A, D112A, Q119A, N205A, S214A, S238A, R280A, and K253A. Left: The PETase structure is shown as a surface model with a gray color. The three residues forming a catalytic triad (S160, D206 and H237) are displayed as green-colored sticks. The PET substrate is shown as yellow-colored sticks. Right: Side view of the substrate binding mode of PETase at the active site. The L-shaped binding cleft, consisting of subsite I, IIa, IIb, and IIc, are indicated in the dash line.

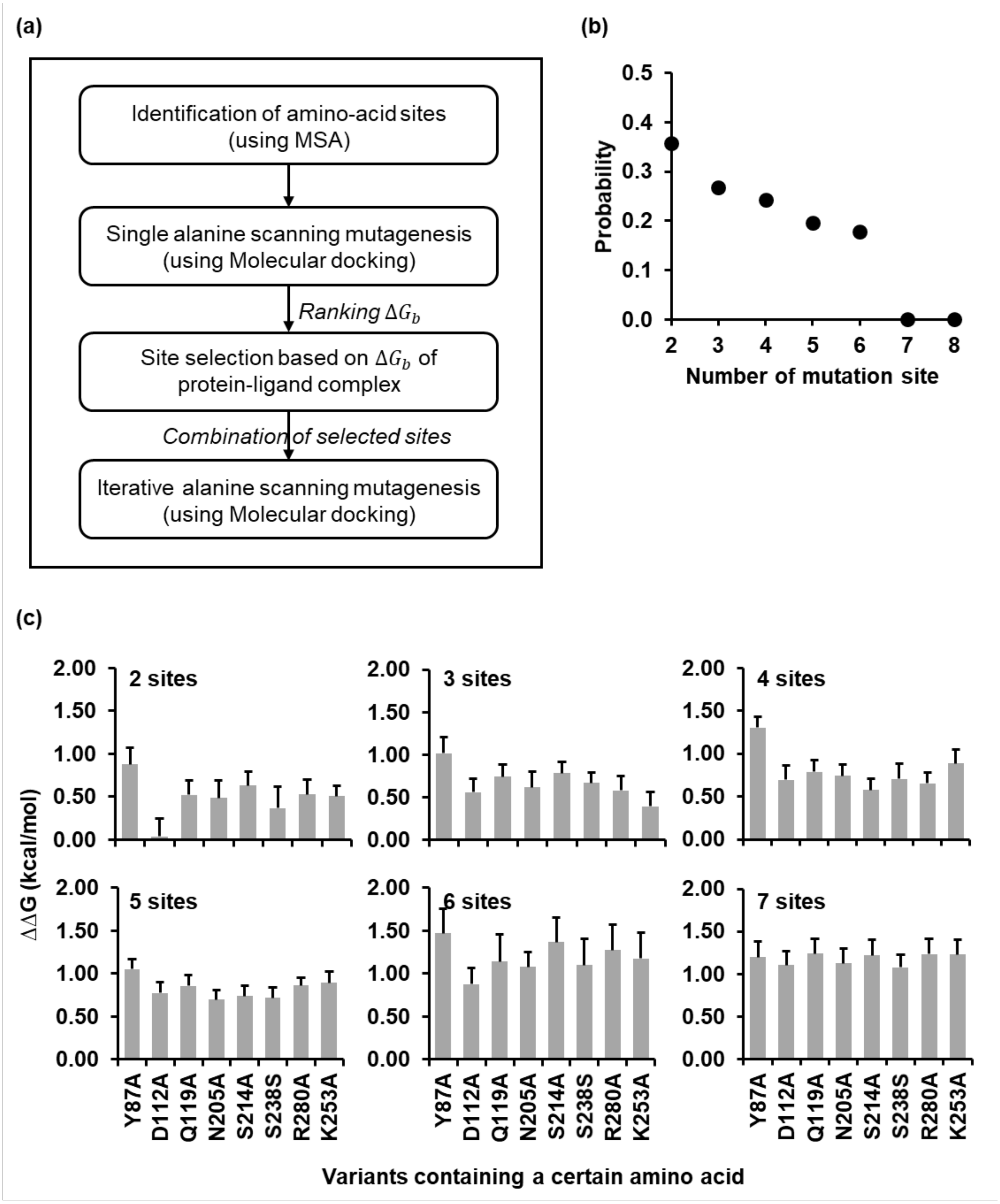

Figure S2. Results of iterative *in silico* alanine scanning mutagenesis. (**a**) Schematic diagram for iterative *in silico* ASM. Each step was performed in the sequencial format. (**b**) The probability of finding desired variants with different combination of mutaion sites. (**c**) The relation of selected amino acid sites and binding free energy with different numbers of mutation sites. The bar graphs show the average binding free energy difference (ΔΔG) of the PETase variant-PET complexes. Data are means ± S.D., where the mean is the average ΔΔG of all variants that contain the labeled amino acid.

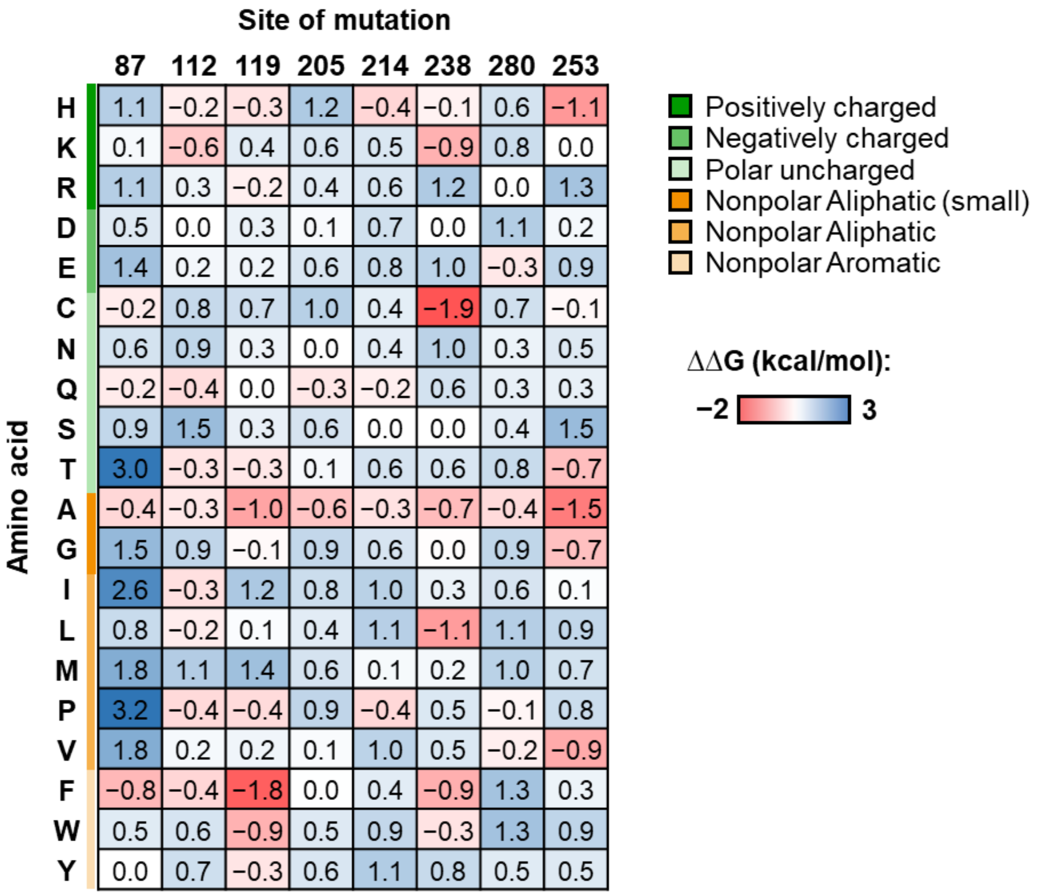

Figure S3. Effect of single site-saturation mutagenesis on the binding free energy. The one-dimension protein sequence space of PETase with different sites of mutation (positions 87, 112, 119, 205, 214, 238, 280, and 253). The color map displays the ΔΔG distribution where the blue color represents high ΔΔG (bad variant), and the red color represents low ΔΔG (good variant).

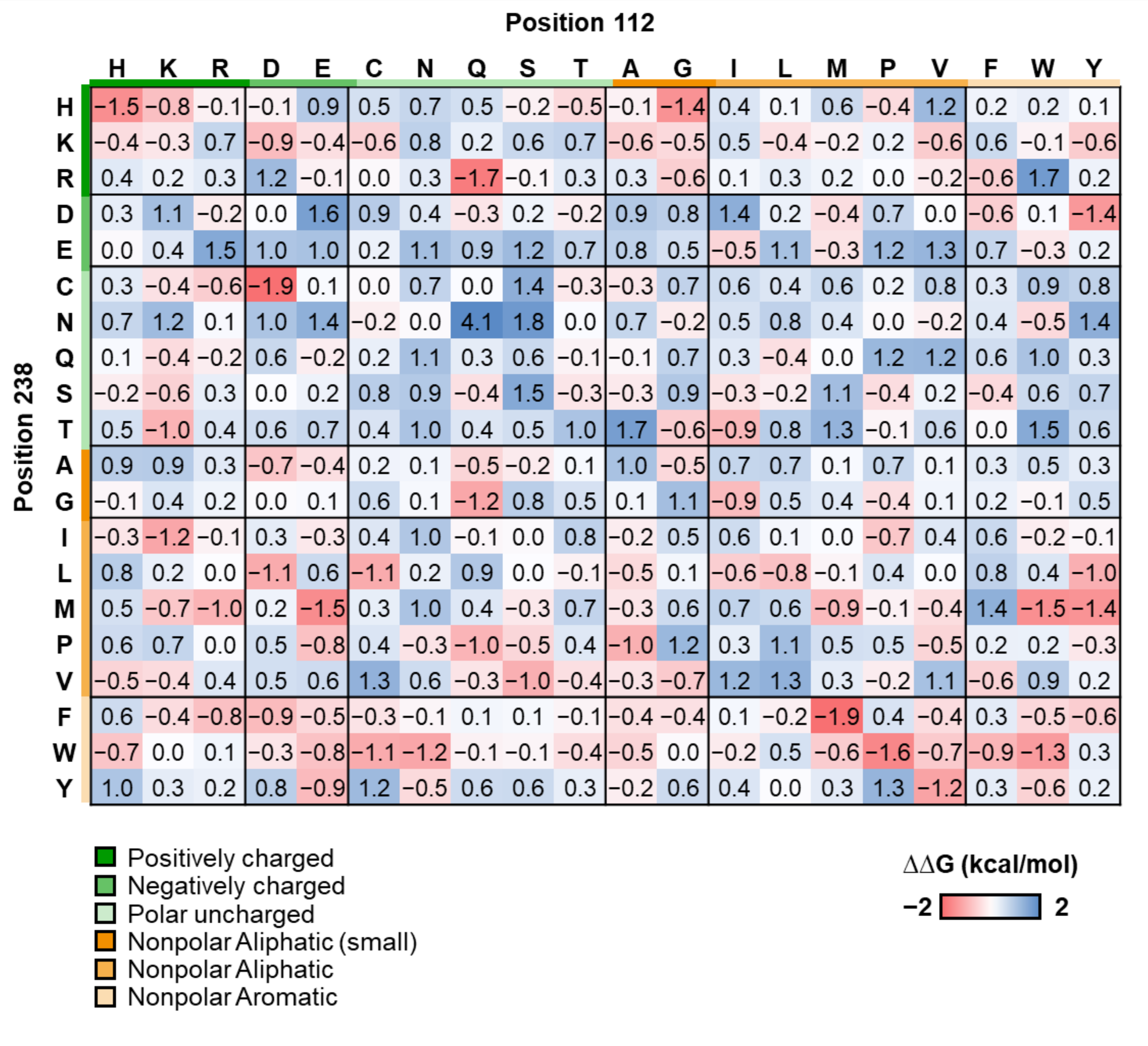

Figure S4. Effect of double site-saturation mutagenesis on the binding free energy. The two-dimension protein sequence space of PETase where the x- and y-axis represent amino acids at positions 112 and 238, respectively. Amino acids are categorized by their physicochemical properties, including positively charged, negatively charged, polar uncharged, nonpolar small aliphatic, aliphatic, and aromatic. In the color bar, the blue color represents high ΔΔG (bad variant), and the red color represents low ΔΔG (good variant).

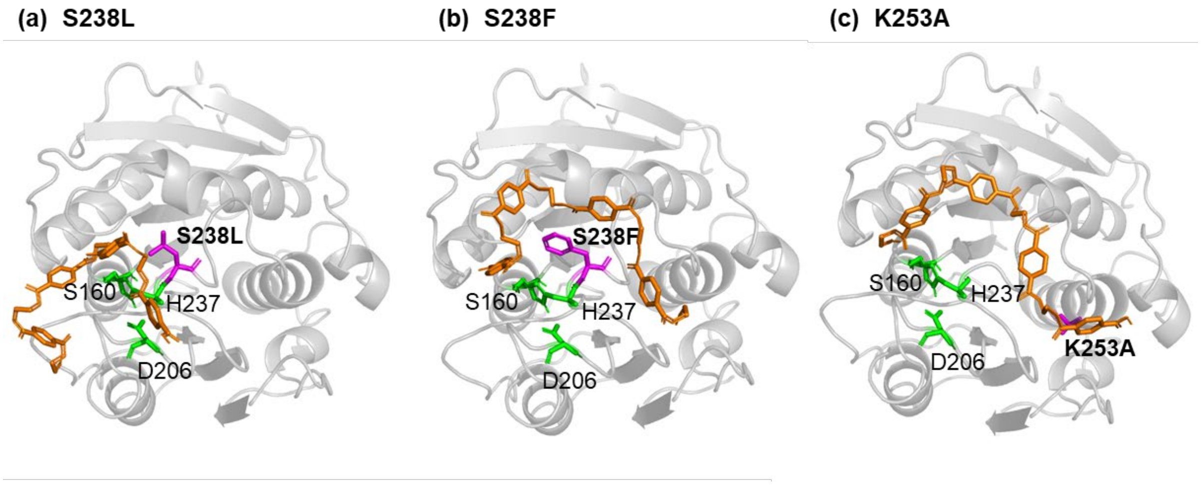

Figure S5. Docking poses of the PETase and PET complex. Three-dimensional ribbon representations of the PETase variants, including (**a**) S238L, (**b**) S238F, and (**c**) K253A, in the complex with the PET substrate were displayed. The catalytic triad, PET substrate docking model, and mutated residues were highlighted in green, orange, and magenta, respectively.

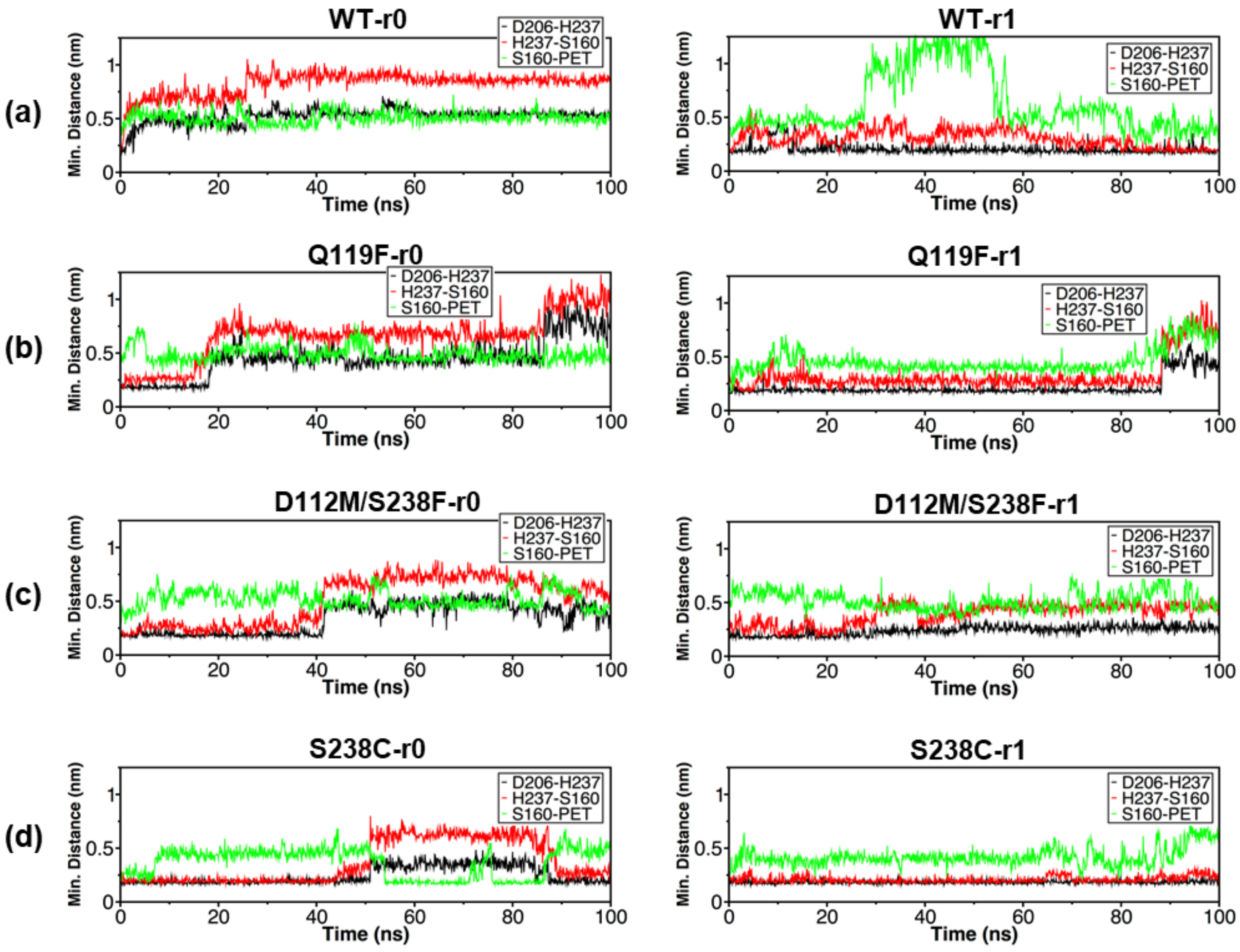

Figure S6. Minimum distances. Minimum distances calculated between the groups of atoms within D206 and H237 catalytic residues (black line), S160 and H237 catalytic residues (red line), and S160 and tetra-PET substrate (green line) are displayed in the following orders of (a) WT, (b) Q119F, (c) D112M/S238F, and (d) S238C with two replicas 'r0' (Left) and 'r1' (Right).

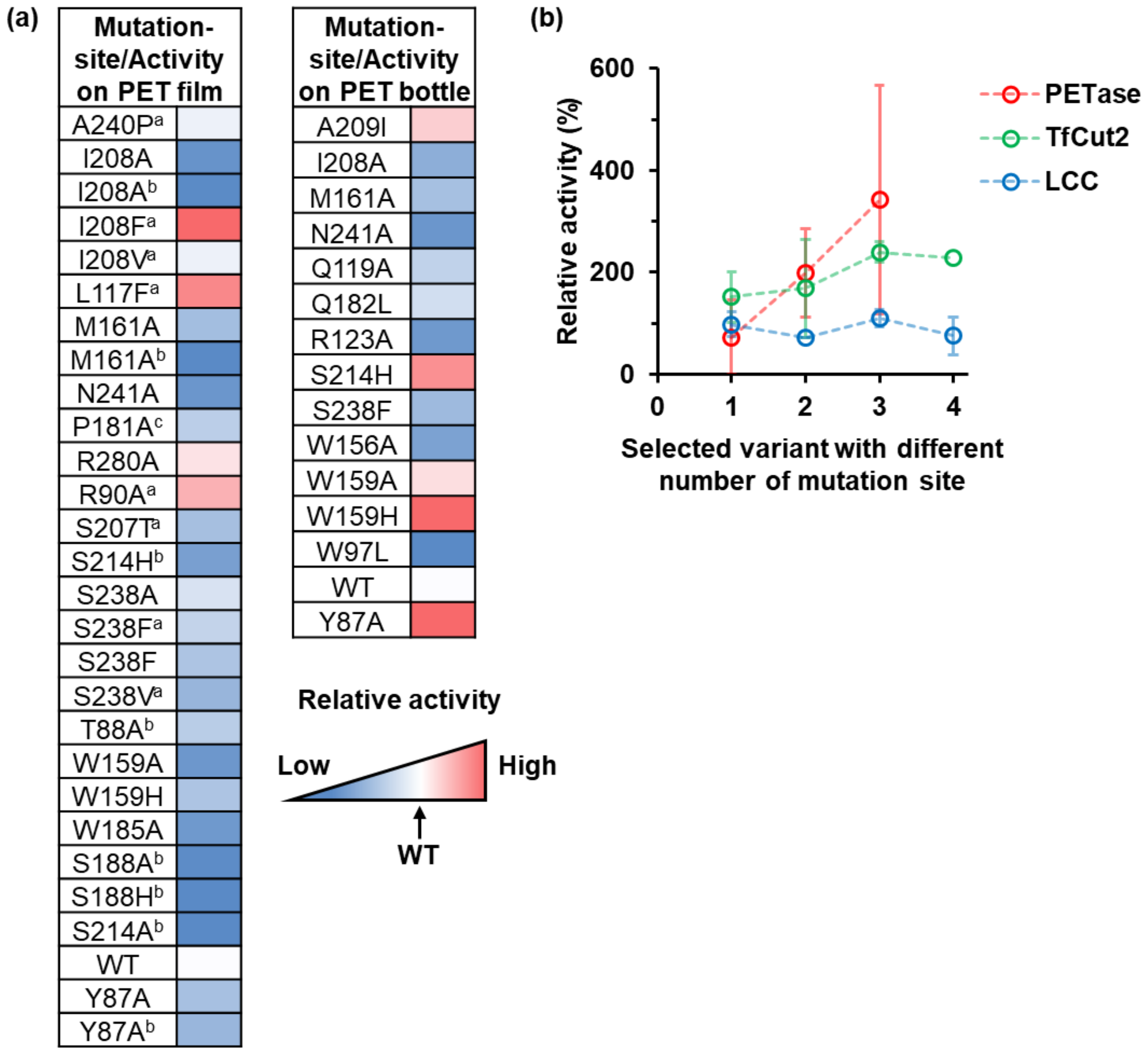

Figure S7. Experimental results of PET-degrading enzymes. (**a**) The PET-degrading activity of PETase variants with different mutation sites were tested either on PET film or in a PET bottle and compared with the wild-type PETase from the experiment. The color bar ranged from blue to red where the blue and red color represented lower and higher PET-degrading activity than the WT (white), respectively. (**b**) The graph shows the relation between the relative activity and the different number of mutation sites of PET-degrading enzymes such as PETase (red), TfCut2 (green), and LCC (blue). Data are mean ± S.D. All data in (A) for PET film (left table) were collected from [23], otherwise indicated, such as [a] [31], [b] [25], and [c] [32]. All data in (A) for PET bottle (right table) were collected from [24].

Table S1. All PETase variants and their mutation sites.

The table shows the alanine substituting combinations of each variant containing one to eight mutation sites (as indicated by the letters A to H) at positions 87, 112, 119, 205, 214, 238, 280, and 253 (as presented in each column). The gray color displays the combination of mutation sites of each variant. Variants that have the binding free energy lower than the WT (negative $\Delta\Delta G$) are highlighted in red rectangles.

Position	87	112	119	205	214	238	280	253
WT								
A1	■							
A2		■						
A3			■					
A4				■				
A5					■			
A6						■		
A7							■	
A8								■
B1	■	■						
**B2**	■		■					
B3	■			■				
B4	■				■			
B5	■					■		
B6	■						■	
**B7**	■							■
**B8**		■	■					
B9		■		■				
**B10**		■			■			
**B11**		■				■		
B12		■					■	
B13		■						■
B14			■	■				
B15			■		■			
B16			■			■		
B17			■				■	
B18			■					■
B19				■	■			
B20				■		■		
B21				■			■	
B22				■				■
B23					■	■		
B24					■		■	
B25					■			■
**B26**						■	■	
B27						■		■
B28							■	■
C1	■	■	■					
C2	■	■		■				
C3	■	■			■			
C4	■	■				■		
C5	■	■					■	
C6	■	■						■
C7	■		■	■				
C8	■		■		■			
C9	■		■			■		
C10	■		■				■	
C11	■		■					■
C12	■			■	■			
C13	■			■		■		
**C14**	■			■			■	

Position	87	112	119	205	214	238	280	253
C15	■			■				■
C16	■				■	■		
C17	■				■		■	
C18	■				■			■
**C19**	■					■	■	
C20	■					■		■
C21	■						■	■
C22		■	■	■				
C23		■	■		■			
C24		■	■			■		
C25		■	■				■	
**C26**		■	■					■
C27		■		■	■			
**C28**		■		■		■		
C29		■		■			■	
C30		■		■				■
**C31**		■			■	■		
C32		■			■		■	
C33		■			■			■
C34		■				■	■	
**C35**		■				■		■
C36		■					■	■
C37			■	■	■			
**C38**			■	■		■		
C39			■	■			■	
C40			■	■				■
C41			■		■	■		
C42			■		■		■	
C43			■		■			■
C44			■			■	■	
C45			■			■		■
C46			■				■	■
C47				■	■	■		
C48				■	■		■	
C49				■	■			■
C50				■		■	■	
C51				■		■		■
C52				■			■	■
C53					■	■	■	
**C54**					■	■		■
C55					■		■	■
C56						■	■	■
D1	■	■	■	■				
D2	■	■	■		■			
D3	■	■	■			■		
D4	■	■	■				■	
D5	■	■	■					■
D6	■	■		■	■			
D7	■	■		■		■		
D8	■	■		■			■	
D9	■	■		■				■

Position	87	112	119	205	214	238	280	253
D10	■	■			■	■		
D11	■	■			■		■	
D12	■	■			■			■
D13	■	■				■	■	
D14	■	■				■		■
D15	■	■					■	■
D16	■		■	■	■			
D17	■		■	■		■		
D18	■		■	■			■	
D19	■		■	■				■
D20	■		■		■	■		
D21	■		■		■		■	
D22	■		■		■			■
D23	■		■			■	■	
D24	■		■			■		■
D25	■		■				■	■
D26	■			■	■	■		
D27	■			■	■		■	
D28	■			■	■			■
D29	■			■		■	■	
D30	■			■		■		■
D31	■			■			■	■
D32	■				■	■	■	
D33	■				■	■		■
D34	■				■		■	■
**D35**	■					■	■	■
**D36**		■	■	■	■			
**D37**		■	■	■		■		
**D38**		■	■	■			■	
**D39**		■	■	■				■
D40		■	■		■	■		
D41		■	■		■		■	
D42		■	■		■			■
**D43**		■	■			■	■	
D44		■	■			■		■
D45		■	■				■	■
D46		■		■	■	■		
D47		■		■	■		■	
D48		■		■	■			■
**D49**		■		■		■	■	
D50		■		■		■		■
D51		■		■			■	■
**D52**		■			■	■	■	
**D53**		■			■	■		■
**D54**		■			■		■	■
D55		■				■	■	■
D56			■	■	■	■		
D57			■	■	■		■	
D58			■	■	■			■
D59			■	■		■	■	
D60			■	■		■		■

Position	87	112	119	205	214	238	280	253
**D61**			■	■			■	■
**D62**			■		■	■	■	
D63			■		■	■		■
D64			■		■		■	■
D65			■			■	■	■
**D66**				■	■	■	■	
**D67**				■	■	■		■
D68				■	■		■	■
D69				■		■	■	■
**D70**					■	■	■	■
**E1**	■	■	■	■	■			
**E2**	■	■	■	■		■		
E3	■	■	■	■			■	
E4	■	■	■	■				■
E5	■	■	■		■	■		
E6	■	■	■		■		■	
E7	■	■	■		■			■
E8	■	■	■			■	■	
E9	■	■	■			■		■
E10	■	■	■				■	■
E11	■	■		■	■	■		
E12	■	■		■	■		■	
E13	■	■		■	■			■
E14	■	■		■		■	■	
E15	■	■		■		■		■
E16	■	■		■			■	■
E17	■	■			■	■	■	
**E18**	■	■			■	■		■
E19	■	■			■		■	■
E20	■	■				■	■	■
E21	■		■	■	■	■		
E22	■		■	■	■		■	
E23	■		■	■	■			■
E24	■		■	■		■	■	
E25	■		■	■		■		■
E26	■		■	■			■	■
E27	■		■		■	■	■	
E28	■		■		■	■		■
E29	■		■		■		■	■
E30	■		■			■	■	■
E31	■			■	■	■	■	
E32	■			■	■	■		■
E33	■			■	■		■	■
E34	■			■		■	■	■
E35	■				■	■	■	■
E36		■	■	■	■	■		
E37		■	■	■	■		■	
E38		■	■	■	■			■
E39		■	■	■		■	■	
E40		■	■	■		■		■
E41		■	■	■			■	■

Position	87	112	119	205	214	238	280	253
E42		■	■		■	■	■	
E43		■	■		■	■		■
E44		■	■		■		■	■
E45		■	■			■	■	■
E46		■		■	■	■	■	
**E47**		■		■	■	■		■
**E48**		■		■	■		■	■
**E49**		■		■		■	■	■
**E50**		■			■	■	■	■
E51			■	■	■	■	■	
**E52**			■	■	■	■		■
**E53**			■	■	■		■	■
E54			■	■		■	■	■
E55			■		■	■	■	■
E56				■	■	■	■	■
F1	■	■	■	■	■	■		
F2	■	■	■	■	■		■	
F3	■	■	■	■	■			■
F4	■	■	■	■		■	■	
**F5**	■	■	■	■		■		■
F6	■	■	■	■			■	■
F7	■	■	■		■	■	■	
F8	■	■	■		■	■		■
F9	■	■	■		■		■	■
**F10**	■	■	■			■	■	■
F11	■	■		■	■	■	■	
F12	■	■		■	■	■		■
F13	■	■		■	■		■	■
F14	■	■		■		■	■	■
F15	■	■			■	■	■	■
F16	■		■	■	■	■	■	
F17	■		■	■	■	■		■
F18	■		■	■	■		■	■
F19	■		■	■		■	■	■
F20	■		■		■	■	■	■
F21	■			■	■	■	■	■
**F22**		■	■	■	■	■	■	
**F23**		■	■	■	■	■		■
F24		■	■	■	■		■	■
F25		■	■	■		■	■	■
**F26**		■	■		■	■	■	■
F27		■		■	■	■	■	■
F28			■	■	■	■	■	■
G1	■	■	■	■	■	■	■	
G2	■	■	■	■	■	■		■
G3	■	■	■	■	■		■	■
G4	■	■	■	■		■	■	■
G5	■	■	■		■	■	■	■
G6	■	■		■	■	■	■	■
G7	■		■	■	■	■	■	■
G8		■	■	■	■	■	■	■
H1	■	■	■	■	■	■	■	■
